# Effects of wood density on mechanical properties of mangrove wood from the Amazon coast

**DOI:** 10.1371/journal.pone.0313824

**Published:** 2024-11-25

**Authors:** Madson Lucas Galvão, Adam Bessa-Silva, Alessandra Silva Batista, Bruno Monteiro Balboni, Iedo Souza Santos, Marcus Emanuel Barroncas Fernandes

**Affiliations:** 1 Laboratório de Ecologia de Manguezal (LAMA), Instituto de Estudos Costeiros (IECOS), Universidade Federal do Pará (UFPA), Bragança, Pará, Brazil; 2 Departamento de Ciências Florestais, Escola Superior de Agricultura "Luiz de Queiroz" (ESALQ), Universidade de São Paulo (USP), Piracicaba, São Paulo, Brazil; 3 Department of Forest and Wood Science, Stellenbosch University, Stellenbosch, South Africa; 4 Departamento de Tecnologia da Madeira, Universidade do Estado do Pará, Belém, Pará, Brazil; Universite Libre de Bruxelles, BELGIUM

## Abstract

Mangrove forests are essential on the Amazon coast, as local communities widely use their wood. However, it is still necessary to understand the mechanical properties of wood typical of mangroves. Our main objective was to understand the influence of density on mechanical properties. Then, we tested the hypothesis that wood density has a stronger influence on the mechanical properties of *R*. *mangle* trees. Five trees of each dominant mangrove species were cut, and the mechanical properties of wood from these species were analyzed according to ASTM D143-14. *Rhizophora mangle* wood presented the highest average values compared to other mangrove species for mechanical properties (*ρ*_*12%*_ = 1031.6 kg m^-3^; *f*_*v0*_ = 21.8 Mpa; *f*_*c0*_ = 79.6 Mpa; *f*_*M*_ = 190.0 Mpa; *E*_*M0*_ = 18.8 Gpa), as well as for resistance and rigidity. Wood from mangrove trees on the Amazon coast has the same trend of mechanical properties as trees from Asian mangroves. *Avicennia germinans* and *Laguncularia racemosa* have a moderate rating. *Rhizophora mangle* stands out for presenting the highest values of these properties, with the species of Rhizophoraceae being considered the most resistant wood among mangrove species worldwide.

## 1 Introduction

Mangrove is an important coastal system that thrives in the transition zone between terrestrial and marine environments, shaped by the tidal regime in tropical and subtropical regions [1]. They are highly productive and have great ecological relevance as they serve as shelter, refuge, and food source for several marine species (invertebrates and vertebrates) and those from freshwater environments, as well as providing coastal protection services [1–4]

Brazil has six typical species of mangrove trees: *Rhizophora mangle* L., *R*. *racemosa* G.F.W. Meyer; *R*. *harrisonii* Leechman, *Avicennia germinans* (L.) L., *A*. *schaueriana* Stapf and Leechman ex Moldenke, and *Laguncularia racemosa* (L.) C. F. Gaertn [5]. The same authors confirm that these species are distributed along the country’s entire coastline, especially along the Amazon coast, where *R*. *mangle*, *A*. *germinans*, and *L*. *racemosa* form the landscape, with *R*. *mangle* being the most dominant species. *Rhizophora mangle* is a dominant tree species in mangrove areas with a higher frequency of flooding, where the waters are brackish or have low salinity [6]. In contrast, *A*. *germinans* trees develop and dominate more saline areas [7], adapting to hypersaline sites where they form monospecific forests with shrubby characteristics, the so-called dwarf *Avicennia* forests [8]. *Avicennia germinans* is the second most dominant species along the entire Amazon coast, followed by *L*. *racemosa* [5], which, in turn, is more common on the margins of mangrove forests and in natural or man-made clearings [6]. It is important to highlight that these species are highly relevant to the coastal communities in the region, being used for multiple functions, such as civil construction (houses, small bridges, stilt houses), weir construction, enclosures for breeding (e.g., poultry, pigs, and cattle), boats, as well as for obtaining firewood and producing charcoal [9, 10]. It is worth noting that the region’s timber forest resources are for subsistence purposes, without established silvicultural practices. This is mainly due to environmental conditions, such as muddy soils and the absence of lianas (vines), which prevents the adoption of forest management techniques, such as selection for thinning, commonly used in other regions, such as Malaysia [11].

Among the characteristics presented by mangrove tree species on the Amazon coast, wood density presents great variation between the three most dominant species, with the highest values being recorded for *R*. *mangle* (0.78 g cm^−3^), followed by *A*. *germinans* (0.64 g cm^−3^), and *L*. *racemosa* (0.57 g cm^−3^) [12]. Density is one of the most relevant variables for evaluating wood, as it relates to other characteristics, such as resistance, stiffness, natural durability, workability, and dimensional stability, among others [13]. Therefore, density is considered one of the main factors in approaching wood quality, both for a better understanding of its ecological functions and to assess its economic value [14, 15]. As a good indicator of the mechanical properties of wood and its behavior, wood density can be used to discuss different ecological issues inherent to mangrove forests, such as the ability of trees to withstand great efforts and environmental pressures regarding their mechanical resistance, when subject to severe weather, such as tropical storms and cyclones [14, 16–18]. In addition, wood density influences the process of trees not yielding to bending or mechanical stress, characteristics that reflect their stiffness [19, 20].

Here, we report the results of evaluating the density and mechanical properties of the three most dominant mangrove tree species on the Amazon coast. The study was carried out on the Caeté-Taperaçu Marine Extractive Reserve (MER), northeast of the State of Pará, where mangrove trees are relevant and have multiple uses for traditional communities [9, 10]. The objective of the present study was to investigate the effect of wood density (*ρ*_*12%*_) on the mechanical properties of mangrove wood, testing its compressive resistance (*f*_*c0*_), resistance to parallel shear to the fibers (*f*_*v0*_), resistance and elasticity to static bending (*E*_*M0*_), and resistance to conventional static bending (*f*_*M*_). Then, we tested the hypothesis that wood density has a stronger influence on the mechanical properties of *R*. *mangle* trees.

## 2 Material and methods

### 2.1. Study area

**Fig 1:**
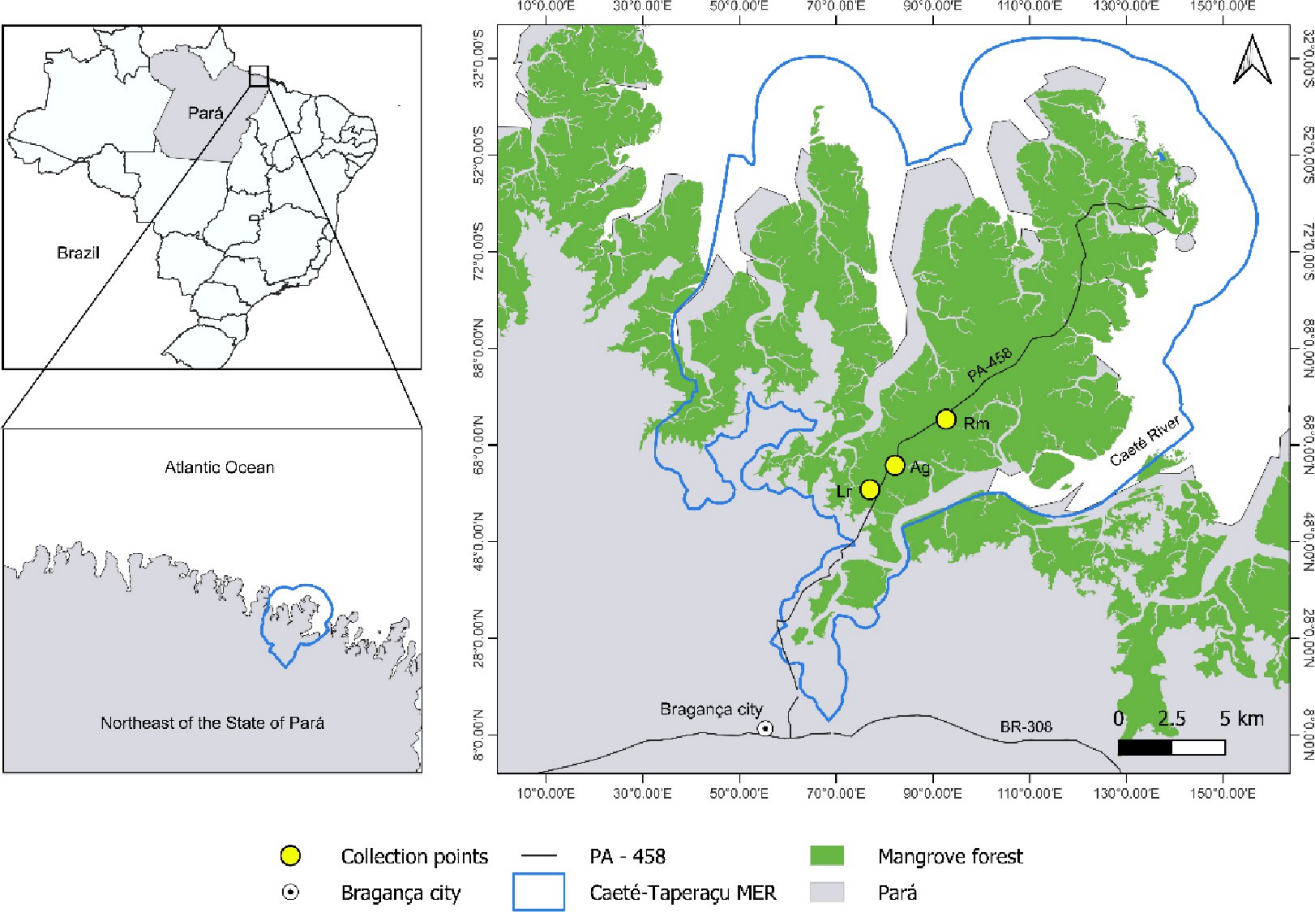
Location of the Caeté-Taperaçu Marine Extractive Reserve, municipality of Bragança, State of Pará, Brazilian Amazon coast. Rm = Forest dominated by *Rhizophora mangle*; Ag = Forest dominated by *Avicennia germinans;* Lr = Forest dominated by *Laguncularia racemosa*. The base map and data were sourced from Instituto Chico Mendes de Conservação da Biodiversidade (ICMBio) and are of free access. The map was created using QGIS software version 2.14.0 (http://www.qgis.org/it/site/).

The study area is located within the Caeté-Taperaçu Marine Extractive Reserve (MER), covers a total area of 42,068,086 ha, and is located on the Ajuruteua peninsula, Bragança, northeastern Pará State (Fig 1). The vegetation cover of the peninsula is approximately 87% mangrove forests (Composed of species: *R*. *mangle*, *R*. *racemosa*, *R*. *harrisonii*, *A*. *germinans*, *A*. *schaueriana*, and *L*. *racemosa*), with soils classified as light or heavy clay [9, 21]. The rest is dominated by salt marshes, *restingas* (coastal sand-dune vegetation), beaches, dunes, and some patches of dry land vegetation [22, 23]. The protected area is under the management of the Chico Mendes Institute for Biodiversity Conservation (ICMBio). The climate in this region is hot and humid, and according to a series of 40 years, the average annual temperature is 26.5°C, with annual precipitation and average relative humidity of 2348.5 mm and 85% [24]. The dry period is from July to November, while the rainy period occurs from December to June [25].

### 2.2. Wood collection

**Fig 2:**
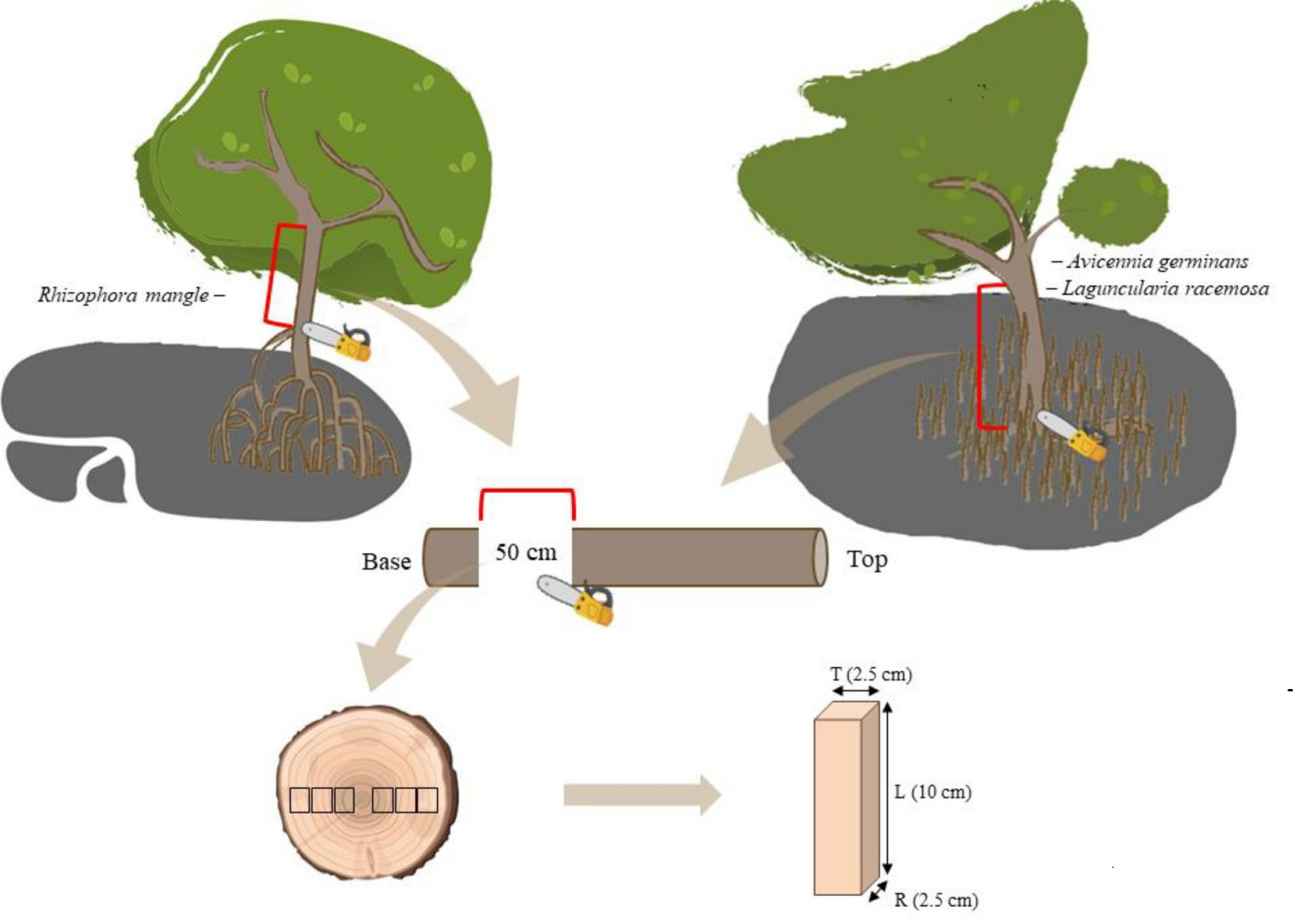
Schematic diagram of wood collection and preparation of specimens for analysis. R = radial; T = tangential; L = longitudinal. Line in red color: log removed from each tree/stem.

Based on previous Diameter at Breast Height (DBH) data from the mangrove forests of Caeté-Taperaçu MER, the criterion for harvesting wood was the median DBH of the forest. The species used were *R*. *mangle*, *A*. *germinans*, and *L*. *racemosa*, with DBH between 20.00–20.24, 23.10–23.89, and 19.00–19.21 cm, respectively. Five species’ trees were harvested per License MMA/ICMBIO/SISBIO n° 77770–1. In the case of *A*. *germinans* and *L*. *racemosa*, the felling was at ground level, and the height of each tree was measured considering the distance between the base and the tip of the felled tree, while the felling for *R*. *mangle* was above the highest rhizophore (Fig 2). Then, a 50 cm long log was removed from the base of each tree and taken to the Mangrove Ecology Laboratory (LAMA) to prepare the test specimens. After preparation, the samples were taken to the Laboratory of Mechanical Testing of Wood and Derivatives (LEMMAD) at the "Luiz de Queiroz" College of Agriculture (ESALQ), University of São Paulo (USP) to carry out mechanical analysis of moisture content (MC).

### 2.3. Measurement of mechanical properties

The wood samples collected in the mangrove forests were processed using a bench circular saw to produce the specimens. Afterward, these test specimens were used to evaluate the density at 12% MC (*ρ*_*12%*_; kg m^-3^) and selected mechanical properties: shear strength parallel to the fibers (*f*_*v0*_; MPa), compression strength parallel to the fibers (*f*_*c0*_; MPa), bending strength (*f*_*M*_; MPa) and stiffness (*E*_*M0*_; GPa), each one of the ten trees collected per species provided between one to three slats with dimensions 28 x 28 x 100 mm, with each slat providing one sample per test. This way, the density value obtained was associated with all samples from the same slat, so relationships between mechanical properties and density could be established at the sample level.

Due to the difficulty of obtaining defect-free samples, the dimensions of the samples were adapted, maintaining, however, their proportions. Table 1 presents the dimensions and number of samples used for each assessment carried out by species. The samples were stored in an acclimatized room with an average temperature of 20°C and a relative humidity of 60% until their mass was stabilized, indicating they reached 12% MC. In sequence, the mass and volume of the samples subjected to parallel compression were measured, and these results were used to determine the density at 12% MC. To assess the mechanical properties, the standard ASTM D143 [26] was used with dimension adaptations mentioned previously. The tests were conducted on a Universal Testing Machine with a capacity of 300 KN.

**Table 1:** Size and number of samples of mangrove trees used to evaluate the density and mechanical properties of the wood in mangrove tree species.

Properties	Dimension	Rm	Ag	Lr
Shear	25 x 25 mm	29	25	12
Compression and density	20 x 20 x 80 mm	27	24	13
Static bending	20 x 20 x 320 mm (280 mm span length)	28	25	13

The higher number refers to the dimension across the wood grain when applicable. Rm = *Rhizophora mangle*, Ag = *Avicennia germinans*, Lr = *Laguncularia racemosa*.

### 2.4. Statistical analysis

The raw data (see S1 Table) were tested for normality (Shapiro-Wilk test) and homoscedasticity (Levene test) with a significance level of α = 0.05 [27]. Since most data violated the assumptions of the parametric model, the Kruskal-Wallis non-parametric test was applied, followed by Dunn’s *post hoc* test. Except for the bending stiffness, ANOVA analysis of variance was used, followed by Tukey´s *post hoc* test [28].

Linear models were built to understand the relationship between wood density and the mechanical properties assessed. The normal distribution of the models’ residues was assessed by comparing the observed with the theoretical quantiles. It also calculated the sample’s specific strength and stiffness, the strength-to-weight ratio (strength: density), and the stiffness-to-weight ratio (stiffness: density). All statistical analysis and graphing were carried out using the free software R [29].

## 3 Results

### 3.1 Density and mechanical properties

The three species showed significant density differences (*H* = 49.79; df = 2; *p* = 1.54 10^−11^), forming three statistical groups (Table 2). *R*. *mangle* was 17% denser than *A*. *germinans* and 42% denser than *L*. *racemosa*. *A*. *germinans*, in turn, was 21% denser than *L*. *racemosa*. The wood of all species presented a coefficient of variation below 20% for density, with *R*. *mangle* and *L*. *racemosa* presenting the lowest values (3.84 and 7.21%, respectively).

**Table 2:** Mechanical properties of wood from mangrove trees on the Brazilian Amazon coast.

Variable	Lr	Ag	Rm
*ρ*_*12%*_ (kg m^-3^)	726.8 ± 52.4 (7.2) c	882.0 ± 88.9 (10.1) b	1031.6 ± 39.6 (3.8) a
*f*_*v0*_ (MPa)	12.5 ± 02.5 (20.0) b	12.1 ± 03.8 (31.3) b	21.8 ± 04.6 (21.1) a
*f*_*c0*_ (MPa)	39.1 ± 06.2 (15.7) c	45.3 ± 07.6 (16.0) b	79.6 ± 06.5 (08.1) a
*E*_*M0*_ (GPa)*	05.8 ± 02.1 (36.0) c	11.5 ± 02.3 (19.6) b	18.8 ± 02.6 (14.0) a
*f*_*M*_ (MPa)	86.4 ± 25.3 (29.3) c	106.6 ± 18.4 (17.2) b	190.0 ± 13.2 (07.0) a

Mean values ± standard deviation and coefficient of variation between parenthesis (%). Lr = *Laguncularia racemosa*; Ag = *Avicennia germinans*; and Rm = *Rhizophora mangle*. *ρ*_*12%*_ (kg m^-3^) = density at 12% MC; *f*_*v0*_ (MPa) = shear strength; *f*_*c0*_ (MPa) = compression strength; *E*_*M0*_ (GPa) = bending stiffness; *f*_*M*_ (MPa) = bending strength; Different letters in the same line indicate significant differences (p<0.05) between mangrove tree species after Kruskal Wallis and ANOVA* test.

Results on the mechanical properties followed the same trend as the density. *R*. *mangle* (the wood with the highest density) presents the highest values compared to the other two species (Table 2). The shear strength was an exception since *A*. *germinans* did not differ from *L*. *racemosa*. However, *R*. *mangle* presented values significantly higher (*H* = 37.16; df = 2; *p* = 1.37 10^−8^) than the other species (74% higher than *L*. *racemosa* and 81% higher than *A*. *germinans*). Likewise, the compressive strength of *R*. *mangle* was significantly higher (*H* = 49.40; df = 2; *p* = 1.88 10^−11^) than that of *A*. *germinans* (75%) and *L*. *racemosa* (103%). On bending, the denser species had higher values for strength (*H* = 53.21; df = 2; *p* = 1.19 10^−11^) and stiffness (ANOVA: *F* = 156.20; df = 2; *p* = 1.43 10^−11^). *R*. *mangle* was 78% and 120% stronger than *A*. *germinans* and *L*. *racemosa*, respectively, and 63% and 224% stiffer than these other two species.

### 3.2. Relationship between density and mechanical properties

**Fig 3:**
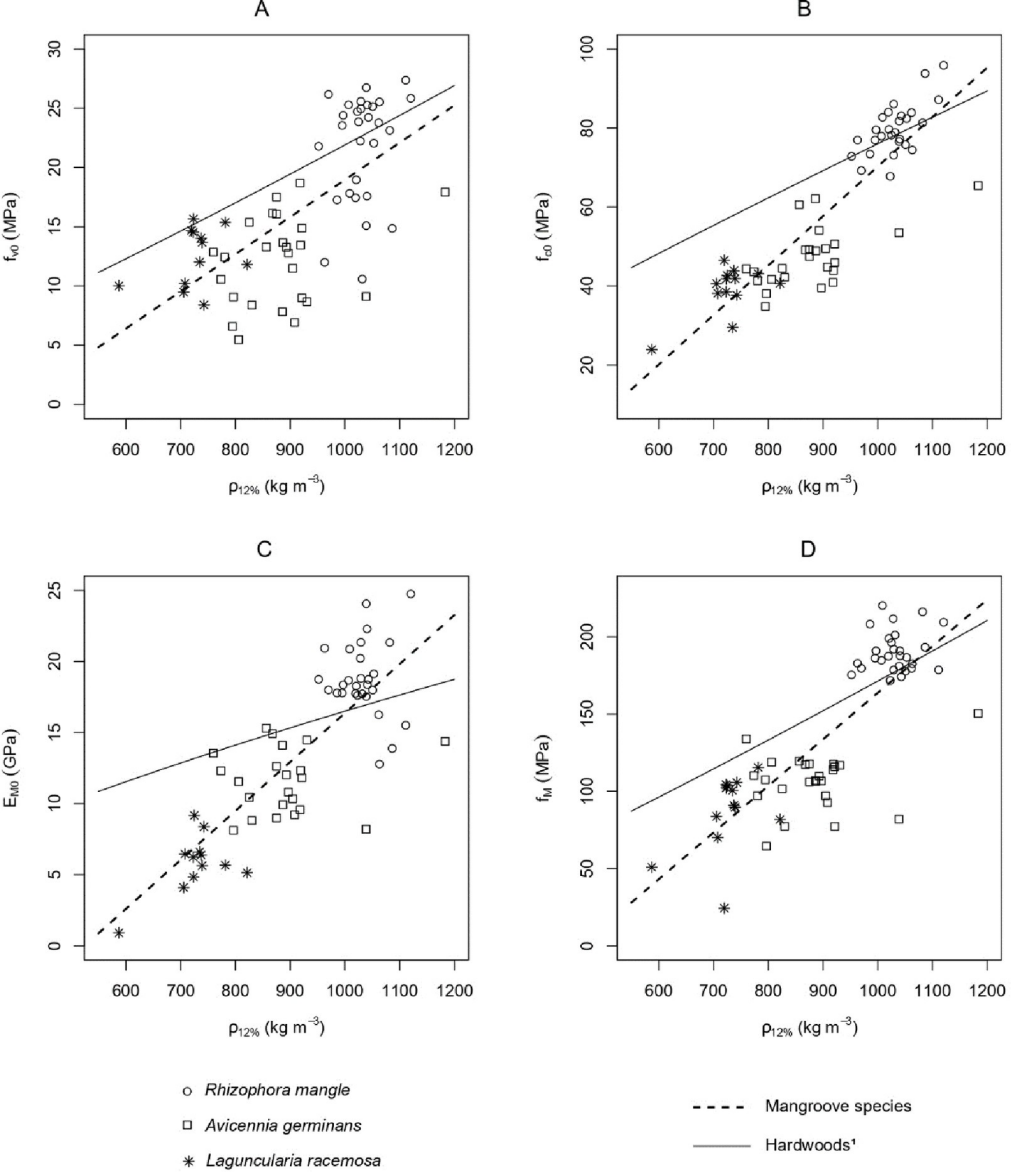
Relationship between wood mechanical properties assessed and density at 12% MC of the three mangrove species *R*. *mangle*, *A*. *germinans*, and *L*. *racemosa*. *ρ*_12%_: density at 12% MC; (A) shear strength (*f*_*v0*_) (B) compressive strength (*f*_*c0*_); (C) bending strength (*f*_*M*_); (D) bending stiffness (*E*_*M0*_). Dashed black lines refer to the linear models from the mangrove species together. Solid black lines the models for hardwoods in general [30].

The relationship between all mechanical properties with density, assessed through the linear models shown in Fig 3, was positive and significant (all *p* < 2 10^−9^). The coefficient of determination ranged from 0.43 up to 0.77, where the lowest R^2^ was obtained for shear strength (Fig 3A), the highest for compressive strength (Fig 3B), and both bending properties intermediate values (0.65) (Fig 3C and 3D). *Rhizophora mangle* was always in the top-right region of the graphs due to its higher density and mechanical properties. *Laguncularia racemosa* samples were commonly in the bottom-left since they have the lightest and weakest wood among the three species assessed. However, bending stiffness is the only property assessed where *L*. *racemosa* and *A*. *germinans* samples are segregated into two-point clouds.

Mangrove timber models:

(A): *f*_*v0*_ = 0.0314 [*ρ*_*12%*_]– 12.43; R^2^ = 0.4349, *p* = 1.726 10^−09^

(B): *f*_*c0*_ = 0.125 [*ρ*_*12%*_]– 55.10; R^2^ = 0.7723, *p* = 2.2 10^−16^

(C): *E*_*M0*_ = 0.034 [*ρ*_*12%*_]– 18.06; R^2^ = 0.6583, *p* = 1.284 10^−15^

(D): *f*_*M*_ = 0.301 [*ρ*_*12%*_]– 137.86; R^2^ = 0.6612, *p* = 2.2 10^−16^

### 3.3. Wood-specific properties (ratio mechanical property: Density)

**Fig 4:**
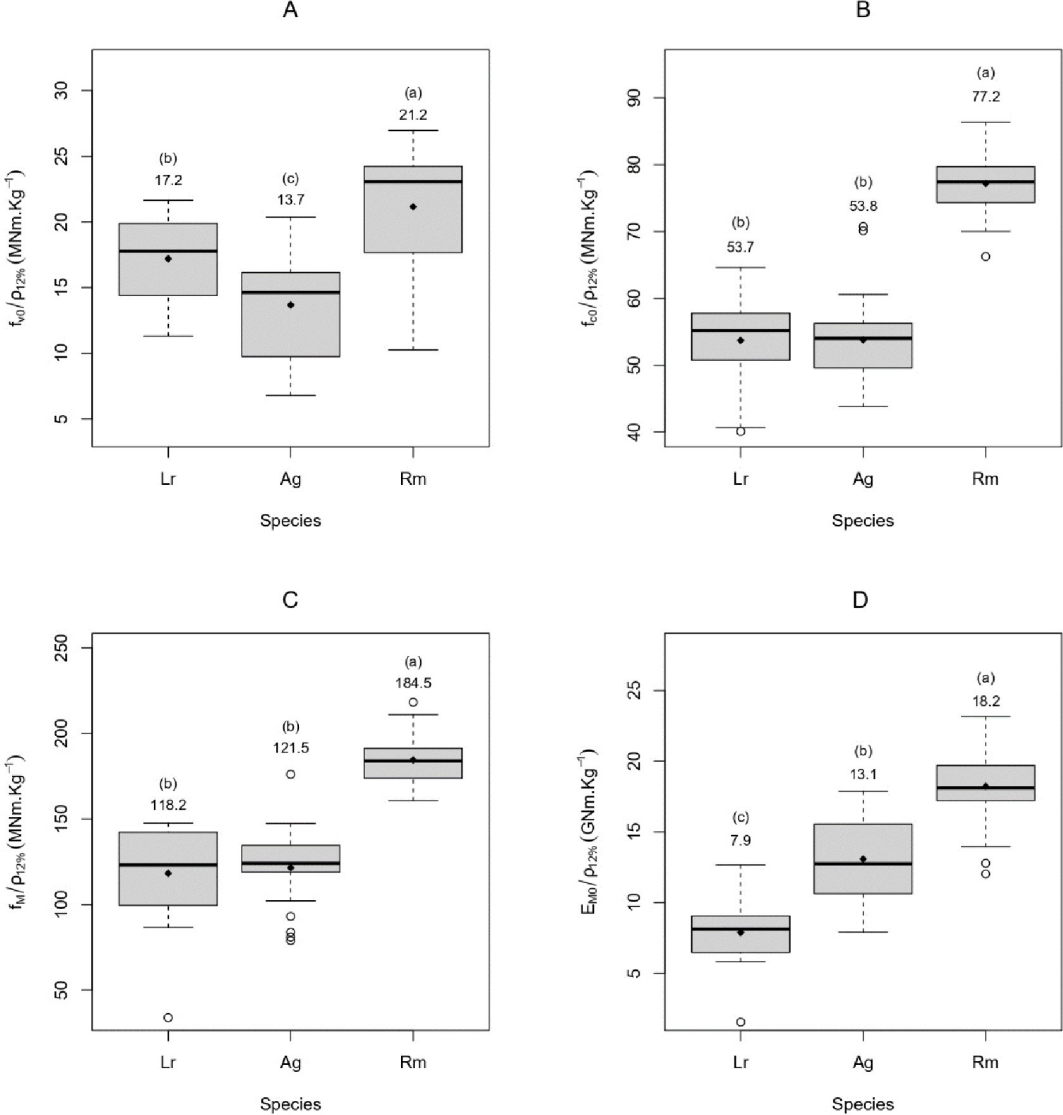
Wood-specific strength (strength to density ratio) and stiffness (stiffness to density ratio) of the three mangrove species *R*. *mangle*, *A*. *germinans*, and *L*. *racemosa*. *ρ*_*12%*_: density at 12% MC; *f*_*c0*_: compressive strength; *f*_*v0*_: shear strength; *f*_*M*_ bending strength; *E*_*M0*_: bending stiffness. Grey boxes represent the second and third quartiles of the data, the horizontal black bar the median, the whiskers 24.65% of the data above or below the second and third quartiles, the empty circles’ values above/below the whiskers, the black diamonds the averages. Letters between parenthesis represent the statistical group (α = 0.05), and the numbers below them are the average.

The mechanical tests hitherto revealed striking differences between the woods of the three mangrove tree species. *R*. *mangle* was the wood with the highest specific properties of the three mangrove species tested. However, the woods of *L*. *racemosa* and *A*. *germinans* did not exhibit significant differences in specific compressive and bending strength (Fig 4). There were three different statistical groups on specific bending stiffness, with *R*. *mangle* having the highest values, followed by *A*. *germinans* and then by *L*. *racemosa*. Under shear, it was *L*. *racemosa*, which had higher specific properties than *A*. *germinans*.

## 4. Discussion

### 4.1. Mechanical of mangrove wood

The wood of mangrove tree species presents wide variations in its mechanical resistance properties. In the present study, we explored these differences by relating these properties to differences in density, as density is one of the most important physical properties and one of the main indicators for classifying the mechanical quality of wood [15, 31–34]. Considering the average density values and according to the classification criteria, *L*. *racemosa* is the species with medium wood, while *A*. *germinans* and *R*. *mangle* with heavy wood [35]. In all results, *L*. *racemosa* wood achieved the lowest mechanical resistance when compared to wood with a higher density, such as *R*. *mangle*, demonstrating the strong and positive correlation between wood density and its mechanical properties [36, 37], considering that density is a characteristic that determines the strength of a tree [36].

Although the trend is that denser woods are also stronger and stiffer [38], these relationships might not be proportional, and the influence of wood density on their mechanical properties may vary even within species [39]. These relationships are assessed through the specific properties, which can be interpreted as the efficiency of using mass for supporting load. As seen in Fig 4, *R*. *mangle* has the higher wood-specific properties among the three mangrove species assessed or the most efficient wood regarding the support of the load. One gram of *R*. *mangle* wood can hold more load than one gram of *A*. *germinans* or *L*. *racemosa* wood. Curiously, *A*. *germinans* was particularly inefficient regarding shear, which might be related to its anatomy. In Fig 3, mangrove models (density x mechanical properties) have higher slopes than generic models for hardwoods [30], this indicates that the wood density in mangrove trees is more important than that of woods from other environments. It seems that there is a combination of a higher efficiency of *R*. *mangle* wood and a low efficiency of *A*. *germinans* and *L*. *racemosa* woods. Fig 3 shows most samples of *R*. *mangle* above the generic hardwood model in all properties (besides compressive strength) and the samples from the other two species below the curve. These differences might be strongly associated with wood anatomy and chemical composition.

**Fig 5:**
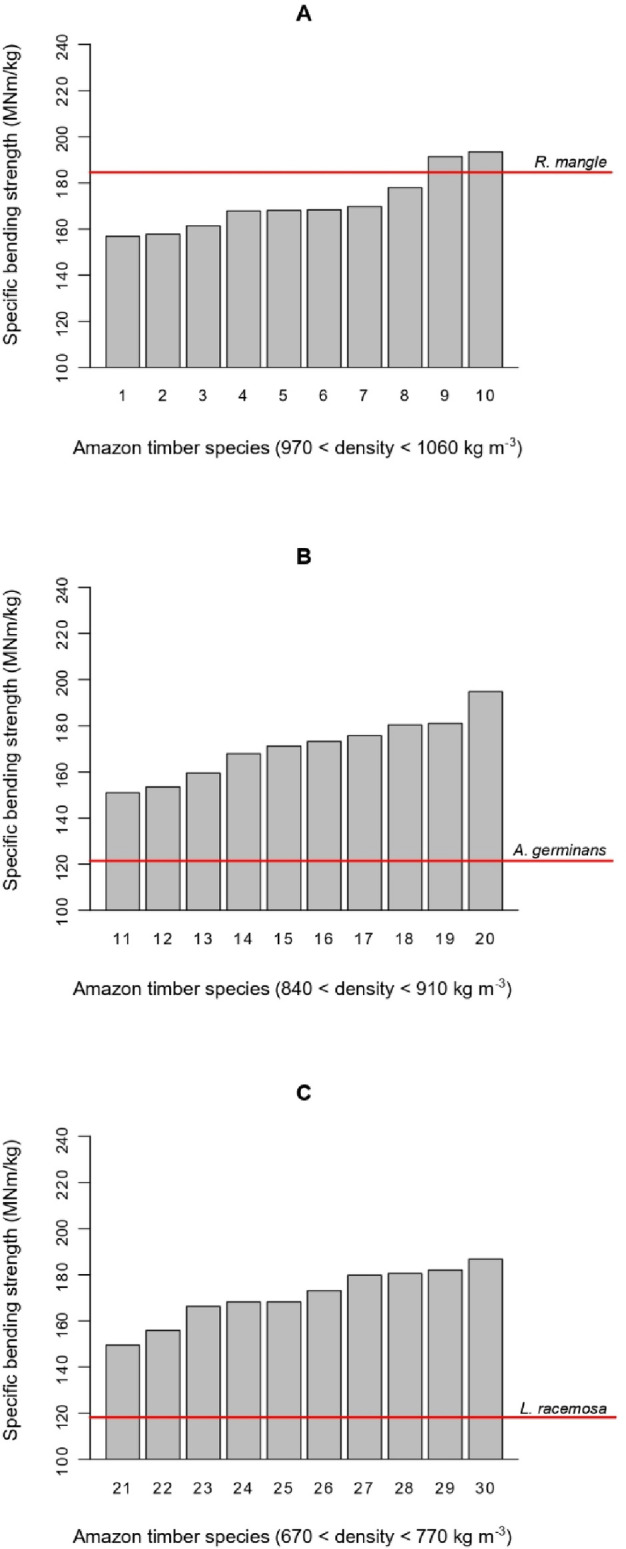
Specific bending strength of *Rhizophora mangle* (A), *Avicennia germinans* (B), and *Laguncularia racemosa* (C), together with ten commercial Amazon timber species of similar density. Source of Amazon timber properties: Brazilian Forest Products Laboratory [40]. List of timber species from 1 to 30 and their properties available on the supplementary material.

Fig 5 plots the wood-specific bending strength of the three species studied and ten commercial Amazon timbers [40] of comparable wood density to each mangrove species. Bending is the main stress trees from mangroves subjected to, especially in strong winds or waves, tropical storms, tornados, etc. While there was no difference in specific bending strength between the three timber density groups, *A*. *germinans* and *L*. *racemosa* are far below the efficiency of Amazon timbers from the same density. Their specific strength was between 62 and 80% of those from the commercial timbers. Since they are, theoretically, more exposed to strong wind and waves than trees from the forest, it would be expected that they have a higher bending efficiency, but this is not the case. This fact might be related to the presence of *R*. *mangle*, a species that is not only very strong under bending, but its specific bending strength is within the top three values of commercial Amazon timbers of the same density. *R*. *mangle* might play an important role in providing mechanical support for the other species, which can specialize their efficiency in other directions, i.e., *A*. *germinans’* high tolerance to salinity. Consequently, *A*. *germinans* and *L*. *racemosa* timber did not need to be strong under bending. In general, these differences, presented in Fig 3 regarding mechanical efficiency, explain the higher slope of the models from mangrove trees compared to hardwoods.

### 4.2. Relationship between mechanical properties and ecological factors

Mangrove trees exhibit varying levels of strength and stiffness, in part due to local environmental factors, such as the higher exposure to wind that these individuals suffer in the coastal region [41]. It is well known that mangrove tree species from the Rhizophoraceae family, especially the genus *Rhizophora*, can efficiently act as a natural barrier against catastrophic phenomena such as hurricanes and tsunamis on the Asian continent [42]. Such phenomena do not occur on the Brazilian Amazon coast and, however, trees of this genus continue to exhibit high levels of wood strength and stiffness, corroborating other studies that point to the fact that trees of the genus *Rhizophora* are not exclusively stronger and stiffer in areas of strong exposure to these meteorological phenomena [41, 43]. However, the strength and stiffness of mangrove tree wood may be closely associated with other ecological factors whose influence appears to occur through extrinsic and/or intrinsic triggers.

Our results show that the mechanical strength of mangrove tree wood follows the trend of these trees dominating mangrove forests on the Brazilian Amazon coast. *R*. *mangle*, for example, is the mangrove tree species that presents the highest values for all mechanical properties analyzed here, followed by *A*. *germinans* and *L*. *racemosa*, this being the same trend for the dominance records of tree species from mangrove forests along the Brazilian Amazon coast [5]. In addition, considering the morphology of the rhizophores, the aerial roots of *R*. *mangle* vary anatomically and allometrically according to their size, supporting a thinner stem with greater mechanical strength when compared to other species. Such characteristics give *R*. *mangle* trees a peculiar strategy to increase the basal area and height of trees in the unconsolidated mangrove soil [44].

The exceptional wood strength and stiffness of mangrove trees, particularly *R*. *mangle*, may be intrinsically linked to their genetic heritage and evolutionary trajectory [45, 46]. Although studies on the genetic basis are scarce and the molecular mechanisms that control this mechanical efficiency are not yet widely characterized, some evaluations of gene expression patterns indicate that specific genes are activated in response to environmental stressors [47, 48]. Thus, analysis of gene expression patterns has already shown the occurrence of genes that have undergone positive selection in the ancestral lineage of species from the Rhizophoraceae family in mangroves [49]. These same authors also suggest that such genes are primarily associated with the responses of their adaptability and regulation to gene expression and that the positive selection of these genes may have been crucial to optimizing the capacity of these species to tolerate environmental stresses and develop adaptive characteristics. This suggests that a significant part of the adaptive processes present in the trees of this family, including those of the *R*. *mangle* species, may be intrinsically linked to their ancestral genetic heritage and subject to modulation [50, 51].

Phylogenetic studies support the idea that the evolutionary lineage of *R*. *mangle* derives from ancestors equipped with highly adaptive characteristics, enabling it to colonize environments with high environmental dynamics [52]. In its broad evolutionary path, the species has likely accumulated a series of genetic mutations, paving the way for the emergence of a range of adaptive traits [53]. Among these traits, the exceptional strength and stiffness of the wood of *R*. *mangle* trees stands out, allowing the species to respond effectively to intense ecological and environmental pressures [54, 55]. Therefore, it is reasonable to postulate that the mechanical efficiency observed in *R*. *mangle* is not an attribute arising solely from recent epigenetic processes. It is plausible that such characteristics are firmly anchored in the species’ evolutionary history, forming an adaptive genetic structure that promotes survival in challenging mangrove environments. However, it is essential to carry out future genomic studies on mangrove tree species, especially in the Rhizophoraceae family, with emphasis on genes related to lignin synthesis–PAL (Phenylalanine ammonia-lyase), C4H (Cinnamate 4-hydroxylase), 4CL (4-Coumarate-CoA ligase), CCR (Cinnamoyl-CoA reductase), CAD (Cinnamoyl-aldehyde dehydrogenase)–which control lignin biosynthesis, providing rigidity and mechanical resistance to the wood.

### 4.3. Relationship between mechanical properties and traditional uses

Mangrove wood, particularly *R*. *mangle*, due to its mechanical strength and rigidity values, is suitable for building houses–mainly for supports, bridges, and fishing pens, something that communities already practice in the region [9, 10]. These mechanical properties of R. mangle ensure that structures made with this wood are durable and resistant to intense physical conditions, such as strong winds, waves, and tides, common in coastal areas [10]. On the other hand, species such as *A*. *germinans* and *L*. *racemosa*, with lower mechanical strength, are used for purposes that require less durability, such as craft tools, firewood, and small fences.

Understanding these mechanical properties is not just an engineering issue. It directly impacts the sustainable management of mangrove forests, as it helps guide the selection of species for different uses while conserving more resilient species such as *R*. *mangle*. By aligning traditional knowledge with scientific data, this research strengthens sustainable practices, allowing these communities to balance their use of mangrove resources while preserving the ecosystem. However, it is important to incorporate local ecological and scientific knowledge to strengthen management, conservation, and stewardship strategies for these forests, thus ensuring the well-being of coastal populations [10].

## 5. Conclusion

Regarding the mechanical properties of mangrove tree species, it was observed that *R*. *mangle* presents higher mechanical strength and wood stiffness values than those obtained for *L*. *racemosa* and *A*. *germinans*. Our results also showed a strong relationship between density and all mechanical properties assessed, but the trend is stronger than those found for other timbers. The same pattern described can be found in other mangrove regions of the planet, where trees from the Rhizophoraceae family stand out in terms of mechanical wood properties compared to other mangrove tree species. These wood mechanical properties described here and the high mechanical efficiency of *R*. *mangle* wood reflect these species’ relevance and ecological role in the mangrove ecosystem dynamics. In addition, our findings greatly contribute as a source of relevant information for management plans, assisting in choosing wood for different uses by traditional communities.

## Supporting information

S1 Table (CSV)
